# Utility and Limitations of the Methyl Diaminobenzene-BODIPY Fluorescent Biosensor for Detecting Methylglyoxal in Cells

**DOI:** 10.1021/acsomega.6c02955

**Published:** 2026-05-28

**Authors:** Rangsima Ponvattanaporn, Jeremy P. Le, Zhimo Li, Ryan J.D. Rivero, Jeffrey Y.W. Mak, David P. Fairlie, Hamish E.G. McWilliam, Abderrahman Hachani, Alexandra J. Corbett, Timothy Patton

**Affiliations:** † Department of Microbiology and Immunology, 534133 University of Melbourne at the Peter Doherty Institute for Infection and Immunity, Melbourne, Victoria 3000, Australia; ‡ Centre for Chemistry and Drug Discovery, Institute for Molecular Bioscience, 1974 The University of Queensland, Brisbane, Queensland 4072, Australia; § ARC Centre of Excellence for Innovations in Peptide and Protein Science, Institute for Molecular Bioscience, The University of Queensland, Brisbane, Queensland 4072, Australia; ∥ Proxima Bio, Melbourne, Victoria 3000, Australia; ⊥ Centre for Pathogen Genomics, The University of Melbourne, Melbourne, Victoria 3010, Australia

## Abstract

Methylglyoxal (MG) is a small and highly reactive byproduct of multiple metabolic reactions. In mammalian cells, MG serves important roles in cell signaling, but elevated levels of MG are toxic and contribute to the progression of many diseases, including autoimmune conditions and cancer. The study of MG is hampered by the lack of simple, accurate, and well-validated detection techniques. Here, we evaluate the previously reported methyl diaminobenzene-BODIPY (MBo) fluorescent probe by testing the detection of MG in solution, cell lysates, and live cells, using both plate reader and flow cytometry methods. We show that the MBo probe detects MG with a low nanomolar limit of detection in PBS and can quantitate low micromolar variations in MG abundance in both live cells and in lysed end point cellular assays. Our results highlight considerations for the use of MBo in common assays, including background assessments when using culture media containing serum, differential sensitivity with a range of lysis buffers, effects of fixation in flow cytometric assays, and depletion of residual MG in tissue culture media. Overall, this study establishes a framework using MBo to accurately assess MG in cellular systems.

## Graphical abstract

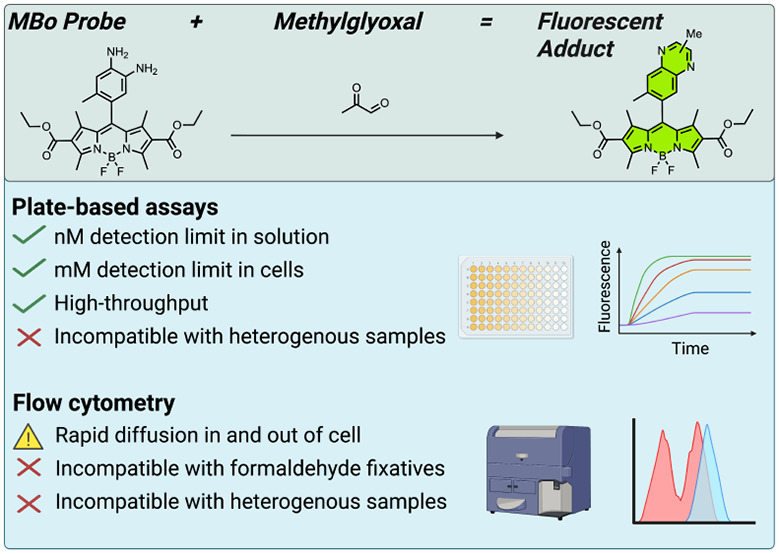

## Introduction

Methylglyoxal (MG), a naturally produced dicarbonyl, is a byproduct of several life-essential cellular metabolic pathways.^1^ MG is highly reactive and forms glycation products with protein residues, basic phospholipids and nucleotides.^2^ Hence, the tight control of MG production and detoxification is critical as MG accumulation contributes to the development of numerous pathological conditions such as diabetes, autoimmune diseases and cancer.^1,3,4^ Research into the pathogenic effects of MG has been hindered by the lack of simple, dynamic and accurate methods to detect and monitor its accumulation in biological systems. The detection of MG is challenging due to its small size, highly reactive nature and low-level abundance in biological samples. While there is a general consensus in estimates for physiological levels of MG between 0.1–2 μM in plasma and between 50–150 μM in tissues,^5–8^ challenges in accurate quantitation are reflected by the high variance in published estimates of human plasma MG, which vary by >1,000 fold at steady state.^8^

Historically, the detection of MG has mainly relied on high-performance liquid chromatography (HPLC), liquid chromatography–mass spectrometry (LC-MS), capillary electrophoresis, and electrochemical methods.^5–7^ The use of these applications is constrained by high instrumentation costs and time-consuming sample preparation, rendering them less suitable for real-time monitoring of MG quantification or high throughput systems. In a cellular environment, MG can react with proteins to form adducts.^9^ While commercial antibodies targeting MG-modified proteins exist, these antibodies are limited in that they nonspecifically recognize protein modification by various molecular species,^10^ and they cannot bind free MG, thus presenting a barrier to its direct and quantitative measurement. The development of fluorescent biosensors to detect MG has garnered interest in the biomedical field,^11^ offering benefits over conventional techniques, due to their rapid reactivity and high sensitivity to low levels of target metabolites.^12^

Over the past decade, multiple chemical probes capable of detecting MG have been synthesized, which are well reviewed.^11^ Of these, diaminobenzene-BODIPY (MBo) was the first to be described,^13^ and remains the most sensitive at a physiological pH.^11^ Here, we provide a detailed evaluation of the MBo fluorescent probe, which exhibits high fluorescence selectivity for MG over other biologically relevant and structurally similar carbonyls,^13^ to detect and quantify MG levels in cellular samples. BODIPY-based probes (such as MBo) are widely used in fluorescence sensing because of their high photostability and tunable optical properties.^14^ The reaction of MG with the *O-*phenylenediamine moiety of the MBo probe induces fluorescence,^13^ and the resulting signal can be detected within defined excitation and emission spectra by flow cytometry and microplate reader instruments.^13,15,16^ Here, we evaluate the application of MBo for the detection of soluble MG levels in solution, cell lysates and live cells, predominantly using THP-1 cells, a monocytic cell line, which is commonly used for biological assays. We have identified key considerations and limitations to the use of MBo as a MG sensor, and optimized experimental conditions to improve MG detection within standard life science protocols. Our results provide a valuable framework to determine dynamic changes in relative and absolute MG. These protocols can be exploited for future investigations of MG in cellular processes, homeostasis, and the development of disease.

## Experimental Section

### Compounds

Methylglyoxal solution ∼40% (w/v) in H_2_O (Cat. #M0252) was purchased from Sigma Chemical Co., MO, USA, and stored at 4 °C.

Methyl diaminobenzene-BODIPY (MBo) was synthesized as previously described,^13^ and stored at −80 °C as a solution in DMSO. The ^1^H and ^13^C NMR spectra matched that reported in the literature (Figure S1).^13^

^1^H NMR (600 MHz, CDCl_3_): δ 6.65 (s, 1H), 6.44 (s, 1H), 4.28 (q, *J* = 7.5 Hz, 4H), 3.57 (br s, 1H), 3.35 (br s, 1H), 2.82 (s, 6H), 1.98 (s, 3H), 1.80 (s, 6H), 1.33 (t, *J* = 7.5 Hz, 6H). ^13^C NMR (150 MHz, CDCl_3_): δ 164.1, 159.1, 147.8, 146.9, 136.7, 133.9, 131.7, 126.7, 124.9, 122.2, 118.8, 115.8, 60.3, 18.6, 15.1, 14.5, 13.2. HRMS *m*/*z* calculated for C_26_H_32_BF_2_N_4_O_4_^+^ [M + H]^+^ 513.2479; found 513.2479.

### Cells

THP-1 (a human monocytic cell line) and M12.C3 (a mouse B cell line) cells were maintained at 37 °C, 5% CO_2_, in complete culture medium (RF10) comprised of Roswell Park Memorial Institute (RPMI, Gibco BRL, Vic., Australia, Cat. #61870127) media supplemented with 10% heat-inactivated fetal calf serum (FCS; Corning, Cat. #35-015-CV), nonessential amino acids (Thermo Fisher Scientific, Cat. #11140076), 80 IU/mL penicillin and 80 μg/mL streptomycin (Gibco, Cat. #15140-122), 2 mM l-glutamine (Gibco, Cat. #25030081), 50 μM β-mercaptoethanol (Sigma, Cat. #M3148), and 5 mM HEPES (Sigma, Cat. #H3375). In cell-based assays, THP-1 cells were washed and resuspended in RF10 without FCS (RF0). Cells were maintained in logarithmic growth phase between 2 × 10^5^ and 1 × 10^6^ cells per mL. For each experiment, viable cells were counted by contrast staining with 0.2% (w/v) Trypan Blue (Cat. #T8154, Sigma Chemical) using a hemocytometer (Cat. #Z359629, Sigma Chemical), under a light microscope. End point viability was determined using the CellTiter-Glo 2.0 Cell Viability Assay (Promega, Cat. #G9241) as per the manufacturer’s instructions, with luminescence measurements taken on the FLUOstar Omega (BMG LABTECH).

### Preparation of Cell Lysates

Lysis buffers were prepared in phosphate buffered saline (PBS): Tween-20 (0.25% v/v), Triton-X (1% v/v), Saponin (0.3% v/v), and NP-40 (0.1% v/v). Cells were resuspended in lysis buffers at concentrations indicated in the figure legends and incubated at RT for 5 min prior to use in assays.

### Plate-Based Assays

Live or lysed THP-1 cells were prepared at 2 × 10^5^ cells per well in RF10 (unless otherwise indicated), in 96-well flat bottom, black polystyrene plates (Sigma, Cat. #CLS3603). Samples were treated with MG doses as indicated, and fluorescence measured in real time, or at end point, following the addition of 5 μM MBo probe. Fluorescence measurements were taken using the CLARIOstar Plus (BMG Labtech), with excitation and emission set at 488/15 nm and 520/20 nm, respectively, unless otherwise indicated. Live cell assays were measured at either end point or in kinetic mode for up to 24 h, recorded using bottom optics using spiral averaging mode with a scan diameter of 6 mm and 106 flashes per well. For live cell assays, samples were maintained at 37 °C in the plate reader for the duration of the readout, without shaking, while lysates and nonbiological samples were maintained at room temperature for the duration of the assay.

### Flow Cytometric Assays

Cells were prepared for flow cytometry direct from passage, or following experimental manipulation and culture as indicated. Cells were washed in PBS supplemented with 2 mM EDTA (FCS-free FACS buffer) and stained with 5 μM of the MBo probe for 1 h, at a density no greater than 1 × 10^6^ cells per 10 μL of stain (minimum staining volume of 50 μL). Cells were then washed once in FCS-free FACS buffer and resuspended in either FCS-free FACS buffer or fixative (0.5% w/v paraformaldehyde, 2% w/v glucose in PBS) for flow cytometric analysis.

### Data Analysis

Graphical presentation of data and statistical analysis was performed using GraphPad Prism version 10.6.1 (GraphPad Software, Boston, Massachusetts, USA). Assay parameters were defined based on previously published methods,^17^ as described below:

*Limit of the blank:* The mean of the blank (PBS) was defined as the average of eight replicates each from three distinct batches. The limit of the blank was then calculated using the following equation:

(Meanoftheblank)×1.645standarddeviationsoftheblank

*Lower limit of detection*: The lower limit of quantitation (LLOD) was extrapolated from the standard curve, using a fluorescence value input calculated from the following equation:

LLOD=Limitoftheblank+1.645standarddeviationsofalowconcentrationsample

*Lower limit of quantitation*: The lower limit of quantitation (LLOQ) was defined as the lowest concentration of MG which delivered a signal above the LLOD with a coefficient of variation (CV) of less than or equal to 15%.

For the calculation of the LLOQ, 156.25 nM of MG was selected as the lowest concentration standard above the limit of the blank.

## Results and Discussion

### Measurement and Quantitation of Free Methylglyoxal in Solution with the MBo Probe

MBo is a BODIPY-based fluorescent probe containing a phenyldiamino motif that was reported to condense with MG to form a fluorescent adduct (Figure 1a), unique among six dicarbonyls tested, including glyoxal.^13^ The MBo probe has been reported to detect MG using a variety of fluorescent methodologies, including flow cytometry, microscopy and plate-based applications.^13,15,16,18^ Here we sought to evaluate the MBo probe’s sensitivity and suitability for high-throughput applications. The MBo probe used in this study was synthesized as previously described.^13^ Its ^1^H and ^13^C NMR spectra were consistent with the original report^13^ (Figure S1), confirming the chemical structure of the MBo probe. To verify the conformity of our MBo probe with previously published parameters,^13^ we assessed the fluorescent excitation and emission spectra of the MBo probe in PBS, with and without MG (Figure 1b,c). The MBo probe incubated with MG emitted robust fluorescence with a peak at 510 nm (Figure 1b), consistent with previously reported parameters.^13^ An excitation scan revealed that maximum fluorescence is elicited by MBo when excited above 490 nm (Figure 1c). However, to avoid overlap in excitation with the detection wavelengths and for consistency with common instrument filter sets, all subsequent experiments were performed with excitation at 488/15 nm and emission at 520/20 nm.

**Figure 1:**
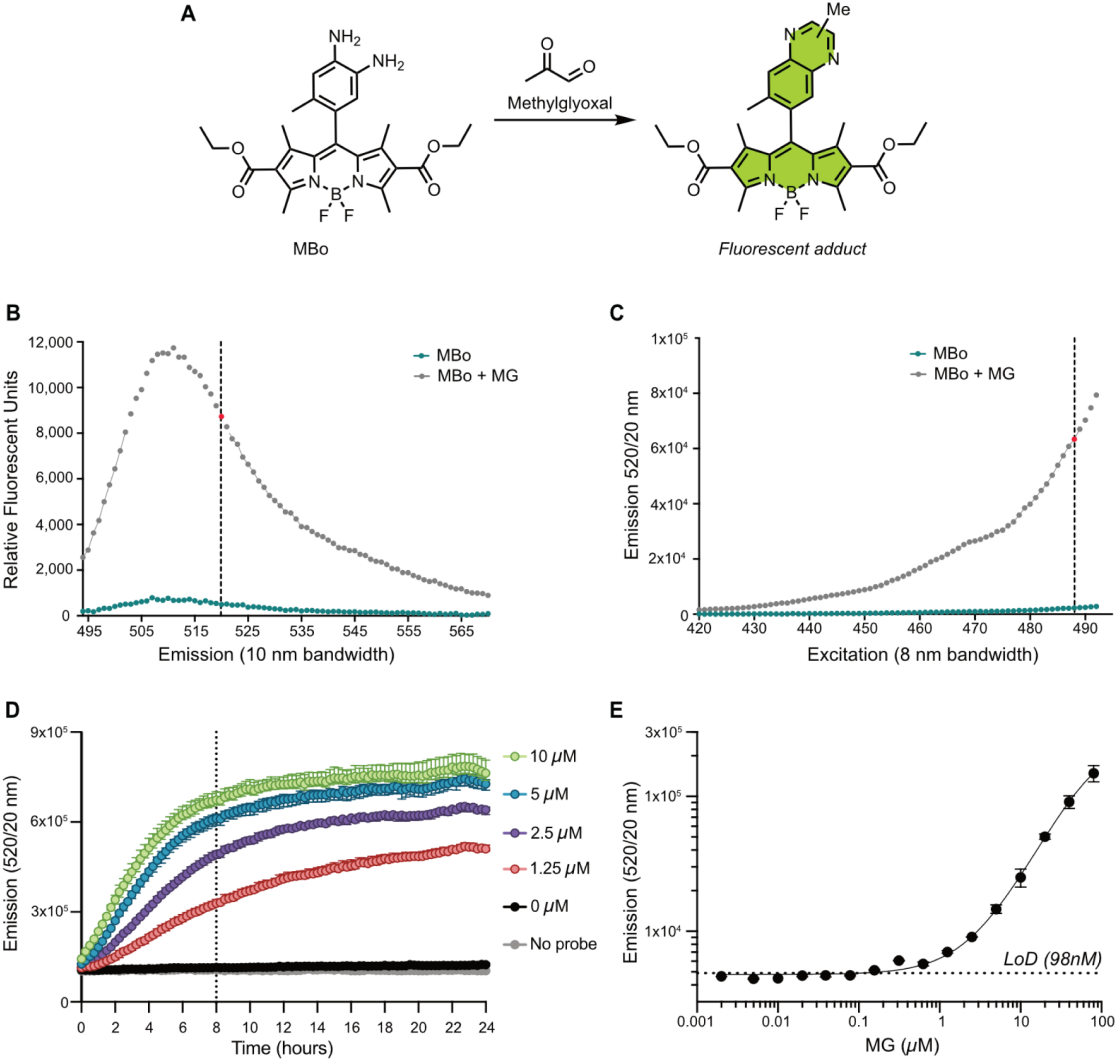
The MBo probe detects nanomolar concentrations of methylglyoxal in solution. (A) Schematic representation of the reaction between methylglyoxal and the *O-*phenylenediamine moiety of the MBo probe, forming a fluorescent quinoxaline adduct. (B) Emission profile of the MBo probe (10 nm bandwidth), with excitation at 470/8 nm and (C) excitation profile of the MBo probe (8 nm bandwidth). Profiles determined from a sample of 5 μM of MBo probe in PBS incubated for 8 h, at RT with 100 μM of MG (gray) or without MG (green). Dashed lines indicate selected centers of excitation and emission filter sets used for subsequent experiments. (D) Time course showing emission (520/20 nm) of the MBo probe (5 μM) over 24 h, treated with a dose titration of MG (colored series), PBS alone (black) and no probe control (gray). Data show the mean and standard deviation of technical triplicates (*n* = 3), with excitation 488/15 nm. (E) Calibration curve showing detection of MG in PBS, in doubling dilutions from 80 μM to 2.4 nM, using the MBo probe following an 8 h incubation at RT with excitation 488/15 nm; emission 520/20 nm.

Previous reports using the MBo probe vary in their protocols, with incubation times for the detection of MG ranging from 1 to 8 h.^13,15,16,18^ Consistent with one previous report,^13^ we found that the fluorescence resulting from the reaction between the MBo probe and MG began to plateau after approximately 8 h incubation, a time point where both the total fluorescence (Figure 1d) and signal-to-noise ratio (Figure S2) were near maximum for concentrations of methylglyoxal between 1 and 10 μM. We next assessed the analytical sensitivity of the MBo probe, based on standardized definitions,^17^ by establishing an MG calibration curve in PBS (Figure 1e). This showed that MBo exhibited nanomolar sensitivity, with a limit of detection at 98 nM (Table 1), consistent with the 50–100 nM range previously estimated, but not analytically defined, in PBS.^13^ These findings demonstrate the capacity of the MBo probe to detect and quantitate low levels of MG, at the lower limit of the reported range in biological fluids such as serum and plasma.^5–7^

**Table 1:** Sensitivity of the MBo Probe for the Detection of Free Methylglyoxal in Solution

Assay parameter	Value
Limit of detection	98 nM
Limit of quantitation	156 nM
Signal to noise ratio	32.7

### MBo Is Suitable for Semiquantitative Analysis of MG in Cell Lysates

We next assessed the detection of MG in cell culture lysates. We sought to develop a simple plate-based end point assay that enables one-step cell lysis and simultaneous detection of intracellular MG. Thus, we tested a variety of lysis buffers to determine the most suitable reagent for detection of MG within cells via the MBo probe. We evaluated the background fluorescence and signal intensity of the MBo probe in the presence of MG using commonly utilized detergents in cell biology. Various detergents and surfactants can be used to disrupt cellular membranes;^19^ however, because MG is a highly reactive carbonyl species potentially prone to oxidation, suitable lysis buffers should be free from oxidizing reagents to avoid interference. We therefore tested Tween-20 (0.25% v/v), Triton-X (1% v/v), Saponin (0.3% w/v), and NP-40 (0.1% v/v), using PBS as control. Among these, Tween-20 exhibited the greatest dynamic range in comparing each lysis buffer alone or spiked with MG (Figure S3a) and was the only buffer capable of detecting exogenous MG spiked in cell lysates (Figure S3a). While the Tween-20 lysis buffer exhibited some reactivity with the MBo probe, this signal was consistently lower than the fluorescence of lysate samples (Figure S3b).

To establish the detection threshold for MG variations within cell lysates, we prepared a calibration curve of MG in THP-1 lysates (in Tween-20) to determine the smallest detectable change in MG levels (Figure 2). In contrast to the detection of free MG in PBS (Figure 1), the reaction between the MBo probe and the lysates spiked with higher doses of MG began to plateau within 4 h (Figure 2a). Indeed, longer sample incubation periods did not enhance detection of MG, but rather resulted in higher backgrounds (Figure S3b,c) and reduced signal-to-noise power in calibration curves (Figure S 3d), with the optimal signal-to-noise ratio observed following 2-h incubation (Figure 2b and Figure S3d). Distinct from the MBo probe’s nanomolar limit of detection in PBS (Table 1 and Figure 1e), the MBo probe could only detect MG variations in the low micromolar range in THP-1 lysates (Figure 2c). Since MG is a ubiquitous byproduct of essential metabolic pathways across species, generating a true negative control allowing the absolute quantification of sensitivity parameters is not feasible.^1,4^ Therefore, the use of the MBo probe in cell lysates should only be considered for semiquantitative comparisons between sample types or for estimating changes in relative MG abundance across treatment groups with the same cell type.

**Figure 2:**
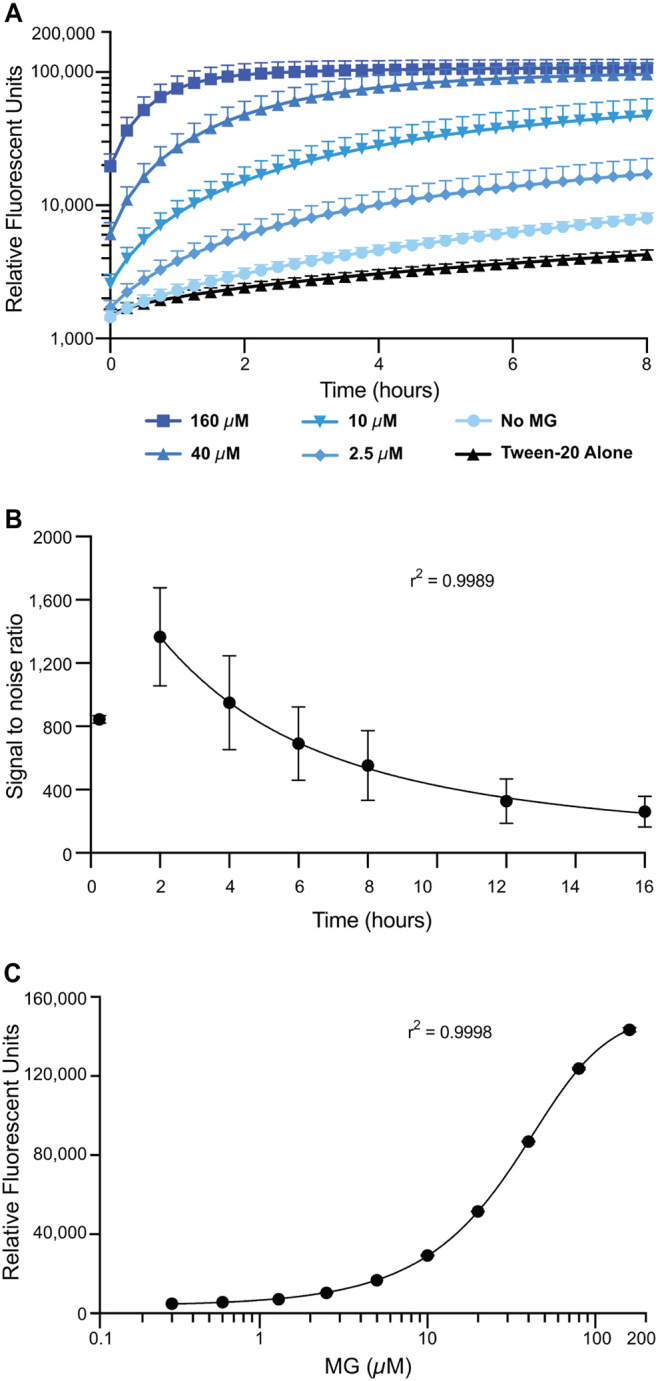
Tween-20 is a suitable lysis buffer for the end-point detection of MG in THP-1 cells. (A) Fluorescent signal of MBo probe in lysates of THP-1 cells spiked with MG. Lysates were spiked with dose titrations of MG at the indicated concentrations (shades of blue), or Tween-20 alone (black). MBo probe (5 μM) added and incubated at room temperature over 8 h with fluorescence readings every 15 min. Points show the mean and SEM of the relative fluorescence intensity (520/20 nm) from four independent experiments (*n* = 4). (B) Signal to noise ratio of lysate spiked with 160 μM MG, against unspiked lysates over 16 h, as in (A). Points show the mean and SEM of the relative fluorescence intensity (520/20 nm) from four independent experiments (*n* = 4). (C) Calibration curve showing detection of MG in lysates of THP-1 cells (as in A), following a 2-h incubation with the MBo probe (5 μM) at room temperature. Data show the mean (A−B) and SD (A−C) of samples prepared in triplicate, representative of four independent experiments (*n* = 4).

### Real-Time Detection of Methylglyoxal in Live Cell Samples

Having established the utility of the MBo probe for end point detection of MG in cell lysates, we next evaluated its suitability for monitoring real time changes in MG levels in live cells. We first assessed the autofluorescence of live THP-1 cells resuspended in PBS in comparison to MBo in PBS and MBo with MG. We found that THP-1 cells in PBS exhibited negligible fluorescence at the excitation/emission wavelength used for MBo detection (Figure S4a).

Next, we considered the use of MBo in assays employing cell samples in tissue culture media. However, incubation of MBo in the standard tissue culture media alone (RF10; containing 10% fetal calf serum [FCS] and other supplements) produced a progressive increase in fluorescence emission over time in the absence of cells (Figure 3a). Unexpectedly, the presence of THP-1 cells in RF10 resulted in a marked reduction in fluorescence over 12 h (Figure 3b) compared to media alone (Figure 3a), likely reflecting cellular detoxification of MG through the glyoxalase system.^20^ To address this limitation, we tested serum-free culture media (RF0: RPMI supplemented with growth nutrients, without FCS) and PBS as alternative media for MG quantification in live cells. Although RF0 elicited slightly higher baseline fluorescence than PBS (Figure 3a,b), background subtraction of probe in each medium without THP-1 cells enabled detection of a cell specific signal over time (Figure 3c). Notably, only RF0 provided a stable and detectable signal between 8 and 24 h (Figure 3c) while also maintaining high cell viability at end point (Figure 3d), thus confirming the suitability of RF0 for extended MG monitoring in cell culture settings.

**Figure 3:**
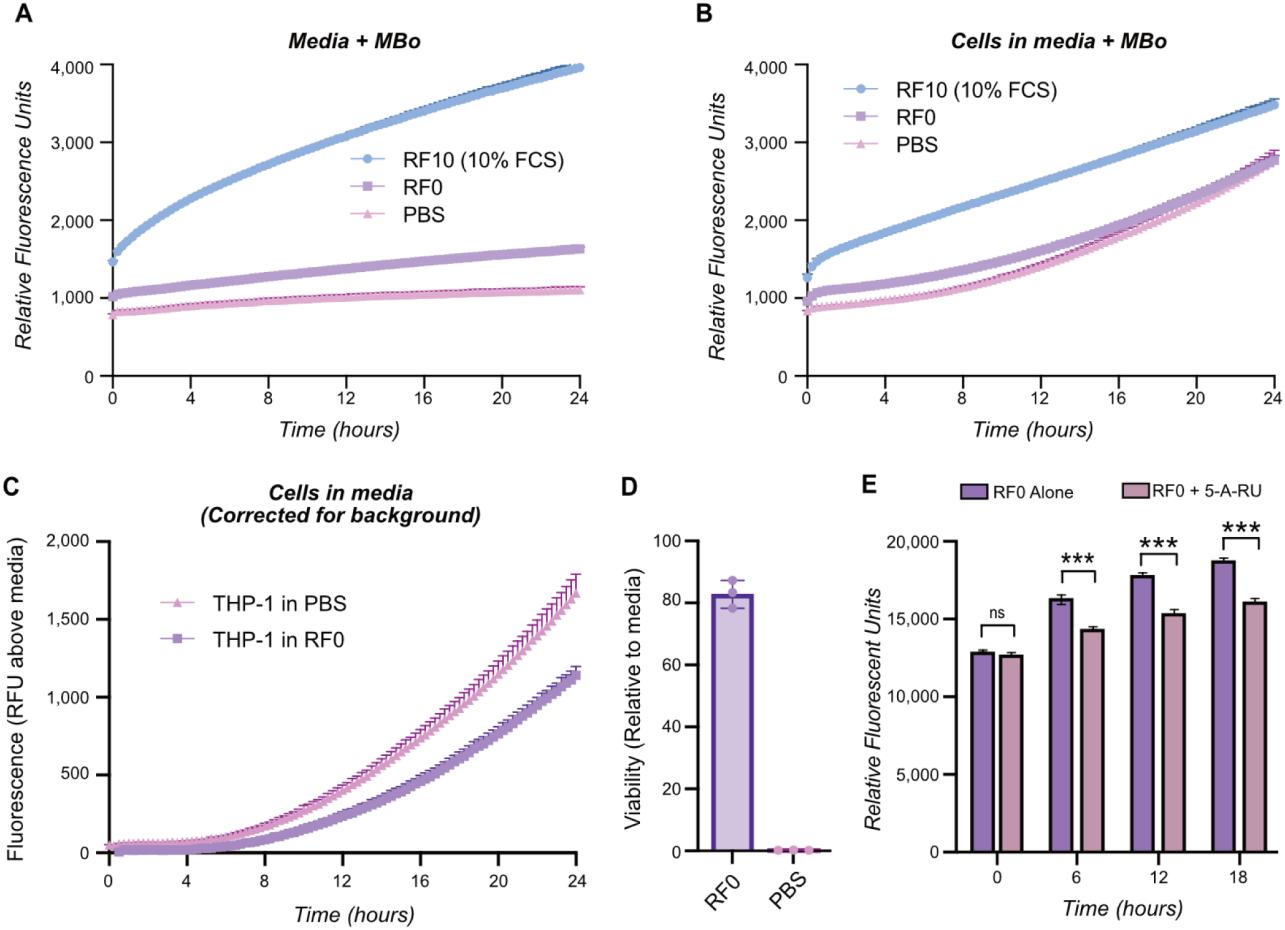
The MBo probe can detect MG in live cells during culture. (A) Real-time fluorescence of 5 μM of the MBo probe in either PBS (pink line), RF0 (purple line) or RF10 (blue line). (B) Fluorescence of the MBo probe when incubated with 2 × 10^5^ cells per well in PBS (pink line), RF0 (purple line) or RF10 (blue line). All samples and cultures (A–B) were maintained at 37 °C and 5% CO_2_, with readings taken every 15 min on a BMG CLARIOstar Plus (excitation 488/15 nm; emission 520/20 nm). (C) Real-time fluorescence of the MBo probe in cultures of THP-1 cells as in (A), corrected for the probe in RF0 or PBS background as in (B), cultured over 24 h, at 2 × 10^5^ cells per well in serum free RF0 media (purple line) or in PBS (pink line). (D) End point viability of THP-1 cells following 24-h incubation in either PBS or RF0 relative to the serum supplement media (RF10) control. Viability quantified on a luminometer, using the CellTiter-Glo 2.0 assay kit. Individual data points represent technical triplicates from a single experiment. (E) Relative fluorescent units (RFU) of the MBo probe over an 18-h time course in RF0 (purple bars) pretreated for 1 h with 100 μM of 5-A-RU. Bars show the mean and SD of triplicates from one experiment, representative of two independent experiments. Statistical significance is presented as not significant (ns), *p* < 0.05 (*), *p* < 0.005 (**), *p* < 0.001 (***); with results from a one-way ANOVA with posthoc *t tests* performed on the indicated groups, correcting for multiple comparisons with the Sidak method.

To verify the requirement for MG in the formation of fluorescent MBo adduct in this system, we depleted MG from serum-free RF0 using 5-amino-6-d-ribitylaminouracil (5-A-RU), a riboflavin pathway intermediate we previously showed is capable of consuming MG via a nonenzymatic condensation reaction.^21,22^ Treatment of RF0 with 5-A-RU significantly and sustainably reduced MBo fluorescence compared to untreated RF0 (Figure 3e), consistent with the reported MBo specificity for MG^13^ and demonstrating the feasibility of endogenous MG depletion in culture medium. However, the same approach did not reduce the MBo signal in live cell samples (Figure S4b); likely due to either the short half-life of 5-A-RU,^23^ the rapid metabolic flux of MG by living cells,^20,24^ or MG having greater reactivity with MBo than 5-A-RU. Collectively, our findings demonstrate that the MBo probe can detect MG in complex biological matrices, including live cells, however assay-specific optimizations are required to validate the probe’s sensitivity in detection of MG flux in distinct experimental conditions.

### Flow Cytometric Applications of the MBo Probe

Flow cytometry is a widely used, high-throughput method for detailed cell phenotyping. Although the MBo probe has previously been applied to detect MG in cultured cells using flow cytometry,^15,16,18^ full validation of the probe for this application is lacking. We therefore sought to consider the stability and detection limits of MBo using typical flow cytometry protocols, which involve multiple wash steps, prolonged staining periods, and often include sample fixation. We found that paraformaldehyde fixation increased overall MBo fluorescence as compared to unfixed cells, while substantially reducing resolution in the detection of exogenous MG (Figure 4a). These findings suggest that the aqueous paraformaldehyde solution used for fixing may react with the MBo probe to produce fluorescent adducts, contributing to the saturation of the signal. Despite these limitations, the probe retained utility for detecting MG in unfixed, uniform mouse and human cell lines, M12.C3 and THP-1 (Figure 4a,b). The probe clearly distinguished 10–100 μM doses of exogenous MG (Figure 4b), although its dynamic range was narrower than that observed in plate-based assays (Figures 1–3).

**Figure 4:**
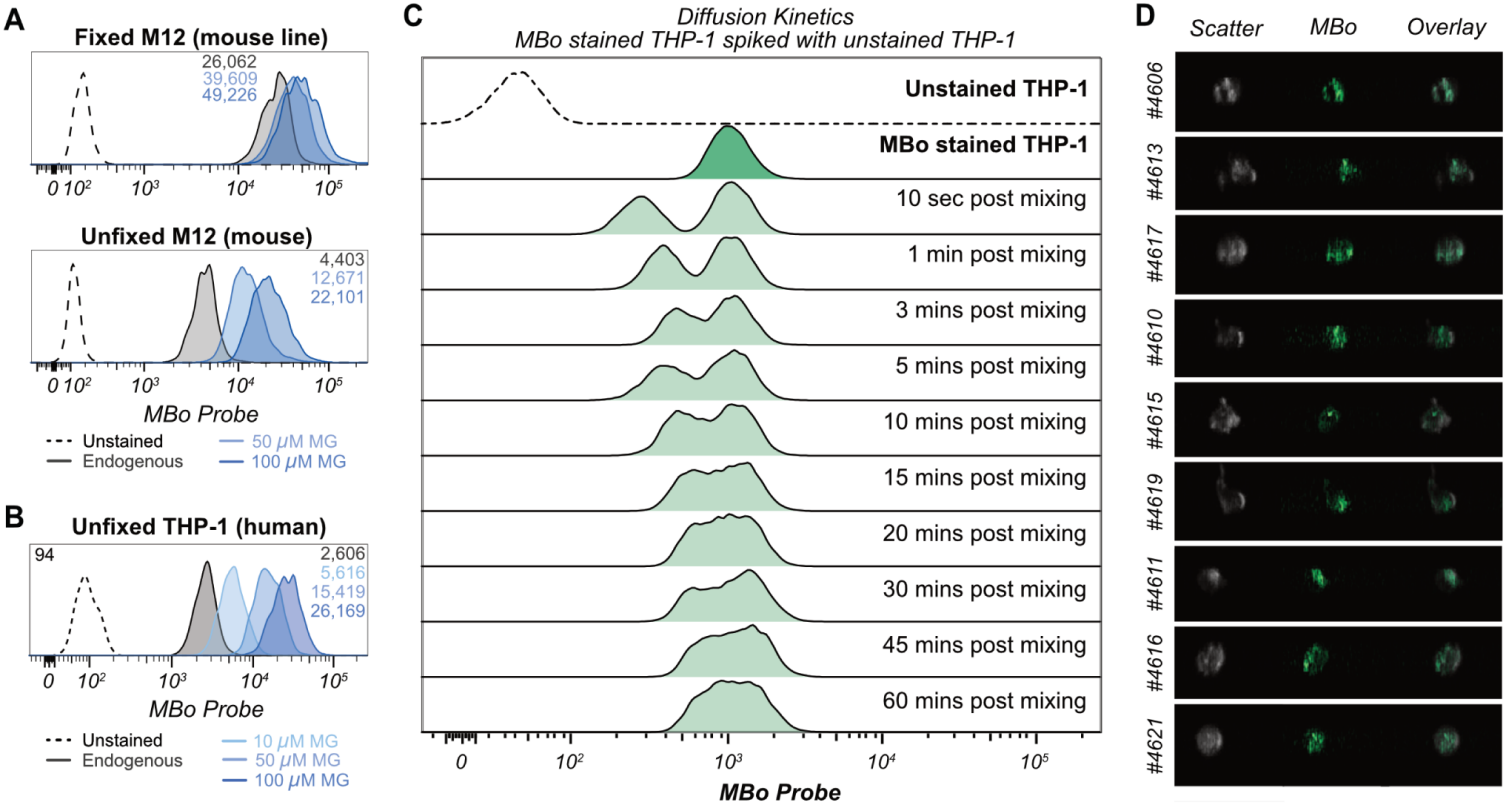
Intracellular detection of MG via flow cytometry using the MBo probe. (A) Fluorescence of the MBo probe in M12.C3 cells with and without fixation. Histograms show detection of endogenous MG in M12.C3 cells (gray shaded histogram) and detection of MG following 1 h treatment with exogenous MG (blue shaded histograms). Numbers shown on the histograms represent the geometric mean fluorescence intensity (gMFI) of each population. (B) Fluorescence of the MBo probe in THP-1 cells, as in (A). (C) Unfixed THP-1 cells were prepared for endogenous detection of MG as described in (A). After staining, sample replicates were mixed 1:1 with unstained cells and incubated for the indicated periods of time before acquiring on the flow cytometer (acquisition time approximately 15 s per sample). (D) Spectral imaging showing MBo probe detection of endogenous MG in THP-1 cells. Images show forward and side scattered light profiles (scatter), fluorescence detection of the probe (MBo), and a semitransparent overlay of both. The nine cells shown are representative of the entire samples (Figure S5C). Samples acquisition performed on a BD Fortessa X-20 benchtop flow cytometer (A–C) and a BD FACS Discover 8 spectral imaging cell sorter (D).

Consistent with the increased signal observed in sera-containing RF10 cell cultures in the plate-based assay (Figure 3b), we found that cells cultured in RF10 similarly exhibited higher levels of MBo fluorescence when compared to those precultured for 2 h in serum-free RF0, in flow cytometry assays (Figure S5a). These findings highlight that the choice of culture medium and serum supplementation also impacts intracellular MG detection in flow cytometric protocols. We next sought to assess the use of the probe in complex and heterogeneous samples. To examine this, we tested the diffusivity of the MBo probe by mixing stained cells with an unstained sample, finding that separation of signal between the positive and negative was rapidly diminished with seconds during sample acquisition (Figure S5b), and completely lost over the course of an hour (Figure 4c). This observation demonstrates a rapid and passive diffusion of the MBo probe across cell membranes, making it incompatible for the analysis of heterogeneous cell samples and cell sorting experiments. Moreover, this may also be a concern for homogeneous cell samples, considering that factors such as sample volume, flow rate, and backflush during sample acquisition; variables which were not evaluated here, would reasonably affect measurements through dilution of the probe.

Finally, to confirm the MBo probe’s diffusivity across the cell membrane, we performed spectral flow cytometry combining real-time imaging to visualize probe localization. THP-1 cells were pretreated with 40 μM MG for 1 h at 37 °C or left untreated, then incubated with MBo for 1 h, prior to image acquisition utilizing a BD FACSDiscover Image S8 at 530 nm. Consistent with our expectations, and with previous microscopy imaging,^13^ the probe was visibly detectable in the cytoplasm of stained cells (Figure 4d and S5c). In summary, despite its specificity for MG and reliable detection of intracellular methylglyoxal in plate-based assays, the MBo probe is not suited to flow cytometric analysis of complex and heterogeneous cell samples due to the rapid rate of membrane diffusion. Future iterations of MG probes may seek to overcome these limitations with chemical “*trapping*” approaches that alter the rate of diffusion across the plasma membrane following the sensor’s reaction with MG.

## Conclusions

A growing body of research has implicated MG in various pathological conditions, but its role remains poorly understood, in part due to the lack of accurate methods capable of detecting this small and highly reactive metabolite. Our study shows that the MBo probe can be employed to detect MG in PBS, cell lysates and live cultured cells with high sensitivity with a microplate reader; while only having limited use for the detection of MG by flow cytometry. The MBo probe enables accurate quantification of MG at nanomolar concentrations in solution. While the probe can also detect low micromolar concentrations of MG in complex biological samples such as live cells and lysates, absolute quantitation is not possible due to the lack of a true negative control. Nonetheless, MBo remains a robust tool for semiquantitative measurements of changes in MG concentration across these sample types.

The application of MBo probe for MG quantification offers several compelling advantages. Plate-based assays and related methodologies are adaptable to high throughput workflows, and remain more cost-effective than HPLC and mass spectrometry-based methods. These attributes underscore the potential of the MBo probe to accelerate research that relies upon scalable cell-based assays. With further optimization, the MBo probe could be extended to more complex biological fluids and multicellular samples, potentially broadening its utility.

## Supplementary Material



## Abbreviations

FCS: Fetal calf serum
LLOD: Lower limit of detection
LLOQ: Lower limit of quantiation
MG: Methylglyoxal
MBo: Methyl diaminobenzene-BODIPY.
