# Supplementary material for: Utility and Limitations of the Methyl Diaminobenzene-BODIPY Fluorescent Biosensor for Detecting Methylglyoxal in Cells
Source: ACS Omega. 2026 May 28;11(22):33104–11. doi: 10.1021/acsomega.6c02955 (PMC13261436; doi:10.1021/acsomega.6c02955)
Supplement: Supplementary file 1 [file ao6c02955_si_001.pdf]

SUPPORTING INFORMATION

Utility and limitations of the methyl diaminobenzene-BODIPY fluorescent biosensor for detecting methylglyoxal in cells

Authors: Rangsima Ponvattanaporn[1], Jeremy P. Le[1], Angela Li[1], Ryan J.D. Rivero[2,3], Jeffrey Y.W. Mak[2,3], David P. Fairlie[2], Hamish E.G. McWilliam[1,4], Abderrahman Hachani[1,5], Alexandra J. Corbett[1,*], Timothy Patton[1,*]

Affiliations

[1]Department of Immunology and Microbiology, University of Melbourne at the Peter Doherty Institute for Infection and Immunity, Melbourne, Victoria, Australia

[2]Centre for Chemistry and Drug Discovery, Institute for Molecular Bioscience, The University of Queensland, Brisbane, Queensland 4072, Australia

[3]ARC Centre of Excellence for Innovations in Peptide and Protein Science, Institute for Molecular Bioscience, The University of Queensland, Brisbane, Queensland 4072, Australia

[4]Proxima Bio, Melbourne, Victoria, 3000, Australia.

[5]Centre for Pathogen Genomics, The University of Melbourne, Melbourne, Victoria, Australia.

Correspondence: Dr Timothy Patton, timothy.patton@unimelb.edu.au, Prof. Alexandra Corbett, corbetta@unimelb.edu.au

Key words: Methylglyoxal, Methyl diaminobenzeneBODIPY (MBo) fluorescent probe, cellular assays, flow cytometry, plate reader

SUPPLEMENTARY FIGURES

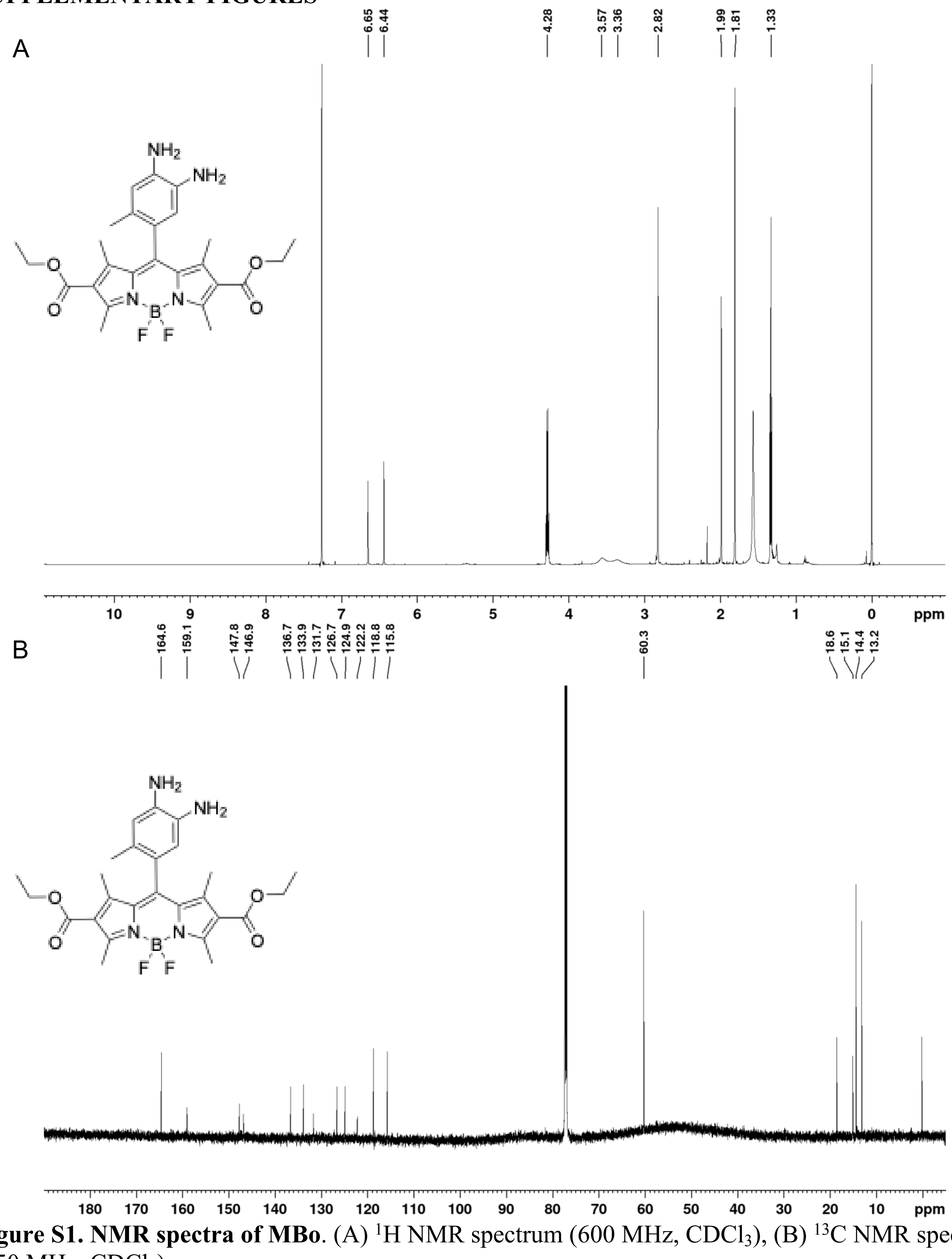

Figure S1. NMR spectra of MBo. (A) ^{1}H NMR spectrum (600 MHz, $CDCl_3$), (B) ^{13}C NMR spectrum (150 MHz, $CDCl_3$).

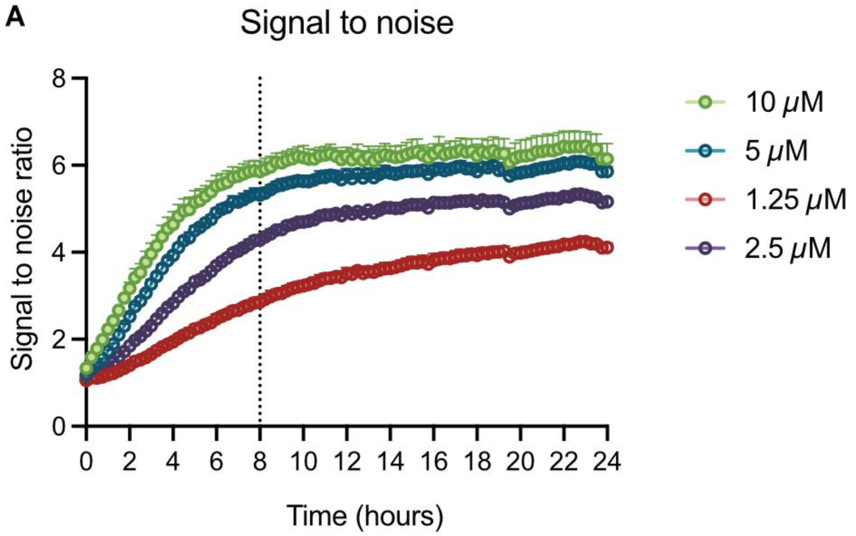

Figure S2. Signal to noise ratio for MG dose titration series as in Figure 1C. The MBo probe (5 μM) was incubated over 24 h with a dose titration of MG (coloured series), and fluorescence (excitation 488/15 nm; emission 520/20 nm) recorded at 15-min intervals. The signal to noise ratio for each dose of MG was calculated as its fluorescence at each time point, divided by the fluorescence of the 0 μM MG control at the respective time point. Data show the mean and standard deviation of technical triplicates (n=3).

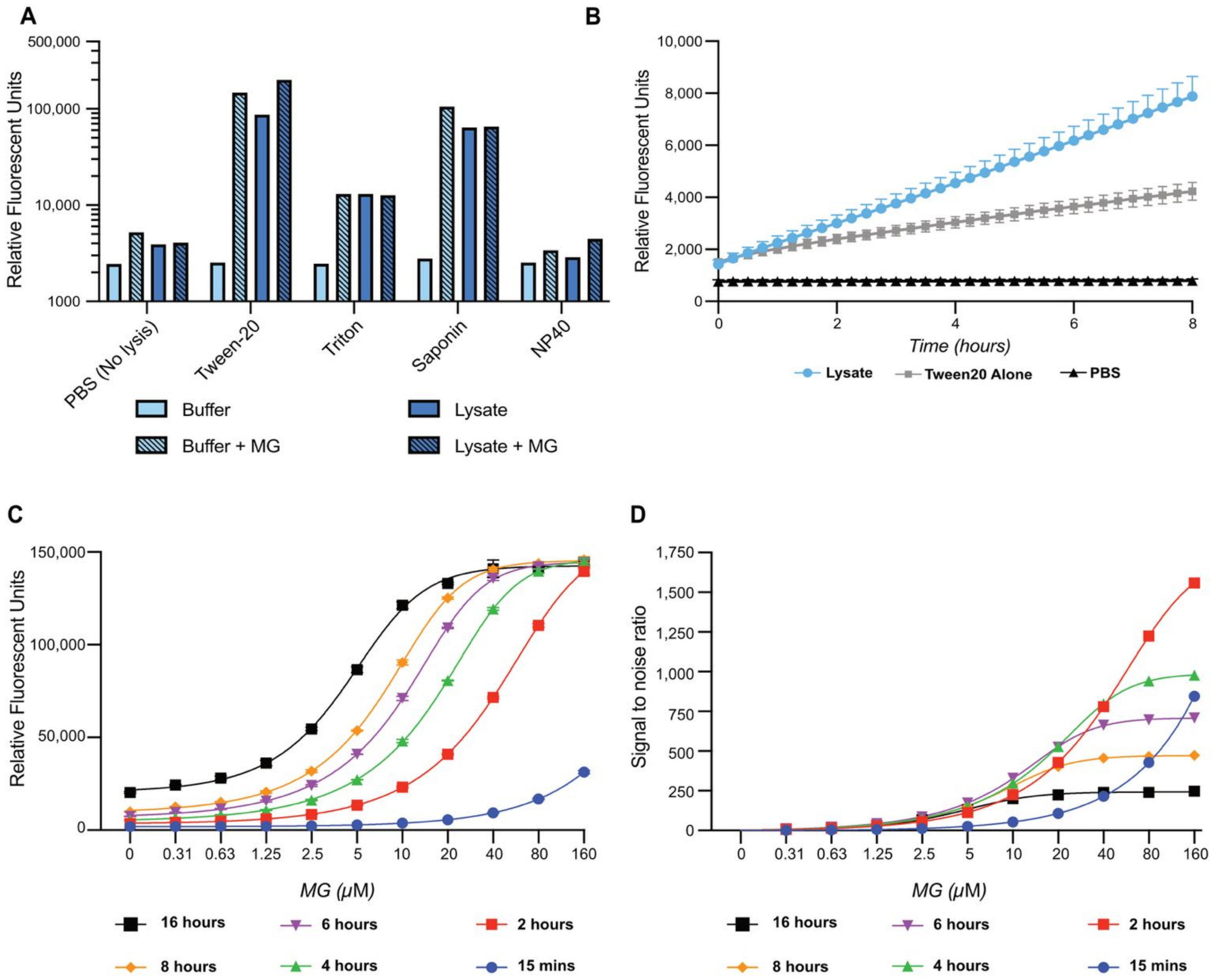

Figure S3. Tween-20 is a suitable lysis buffer for the end-point detection of MG in THP-1 cells (A) THP-1 cell lysates (5×10^5 cells per well) were prepared with either Tween-20 (0.25% v/v in PBS), Triton-X (1% v/v in PBS), Saponin (0.3% v/v) in PBS, NP-40 (0.1% v/v in PBS) or PBS alone. Samples were treated with 10 μM MG for 5 min, before addition of 5 μM MBo and further incubation for 8 h. Bars show individual measurements for lysates (dark blue), lysis buffer alone (light blue) with exogenous MG (hashed bars) or without exogenous MG (solid bars). Fluorescence measurement (Excitation 488/15; emission 520/20 nm) was determined on a Clariostar PLUS plate reader. **(B)** Time series showing fluorescence accumulation of THP-1 lysates prepared in Tween-20 (light blue), or Tween-20 alone (grey), against the background of PBS alone (black). Preparation of lysates and quantitation of RFU as in (A). Data show the mean and standard deviation of triplicates from one experiment, representative of three independent experiments (n=3). **(C)** Calibration curves of MG in THP-1 lysates prepared in Tween-20 as in (A), constructed following incubation with the MBo probe (5 μM) at the indicated time intervals ranging from 15 min to 16 h. **(D)** Signal to noise ratio calculated for the calibration curves shown in (C), calculated by dividing the RFU for each concentration of MG by the lysate background (0 μM, exogenous MG), at its respective time point. Data shows the mean and standard deviation of triplicates from one experiment, representative of three independent experiments (n=3).

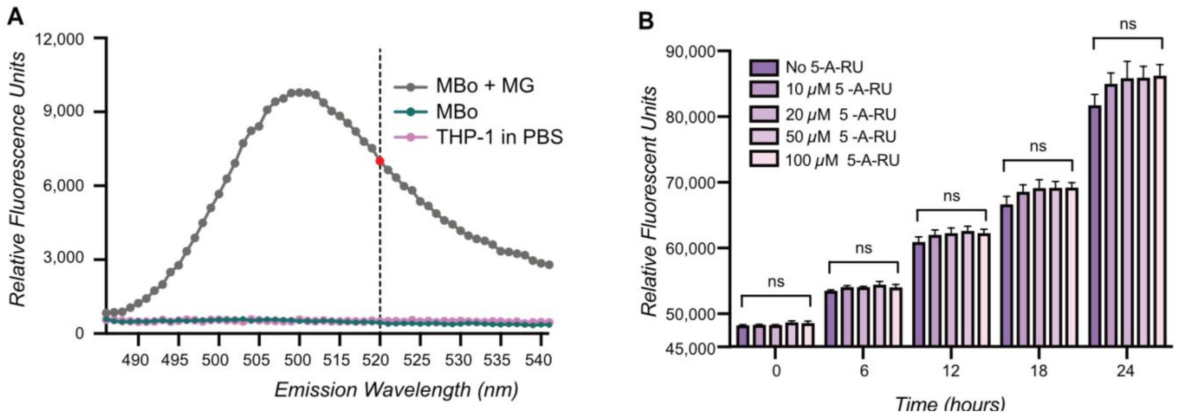

Figure S4. The MBo probe exhibits high backgrounds in media supplemented with FCS (A) Emission profile of 5 × 10^5 THP-1 cells in PBS (pink), 5 µM MBo probe (green), or 5 µM MBo probe with 100 µM MG in PBS (grey). Detection performed following an 8-h incubation with excitation at 488/15 nm and emission 520/20 nm. **(B)** Relative fluorescent units (RFU) of the MBo probe over a 24-h time course with THP-1 cells in RF0 (dark purple bars), pre-treated for 1 h with a dose titration of 5-A-RU (light shaded bars, as indicated). Bars show the mean and SEM of triplicates from one experiment. Statistical significance is presented as not significant (ns), $p < 0.05$ (*), $p < 0.005$ (**), $p < 0.001$ (***); with results from a one-way ANOVA with post-hoc *t-tests* performed on the indicated groups, correcting for multiple comparisons with the Sidak method.

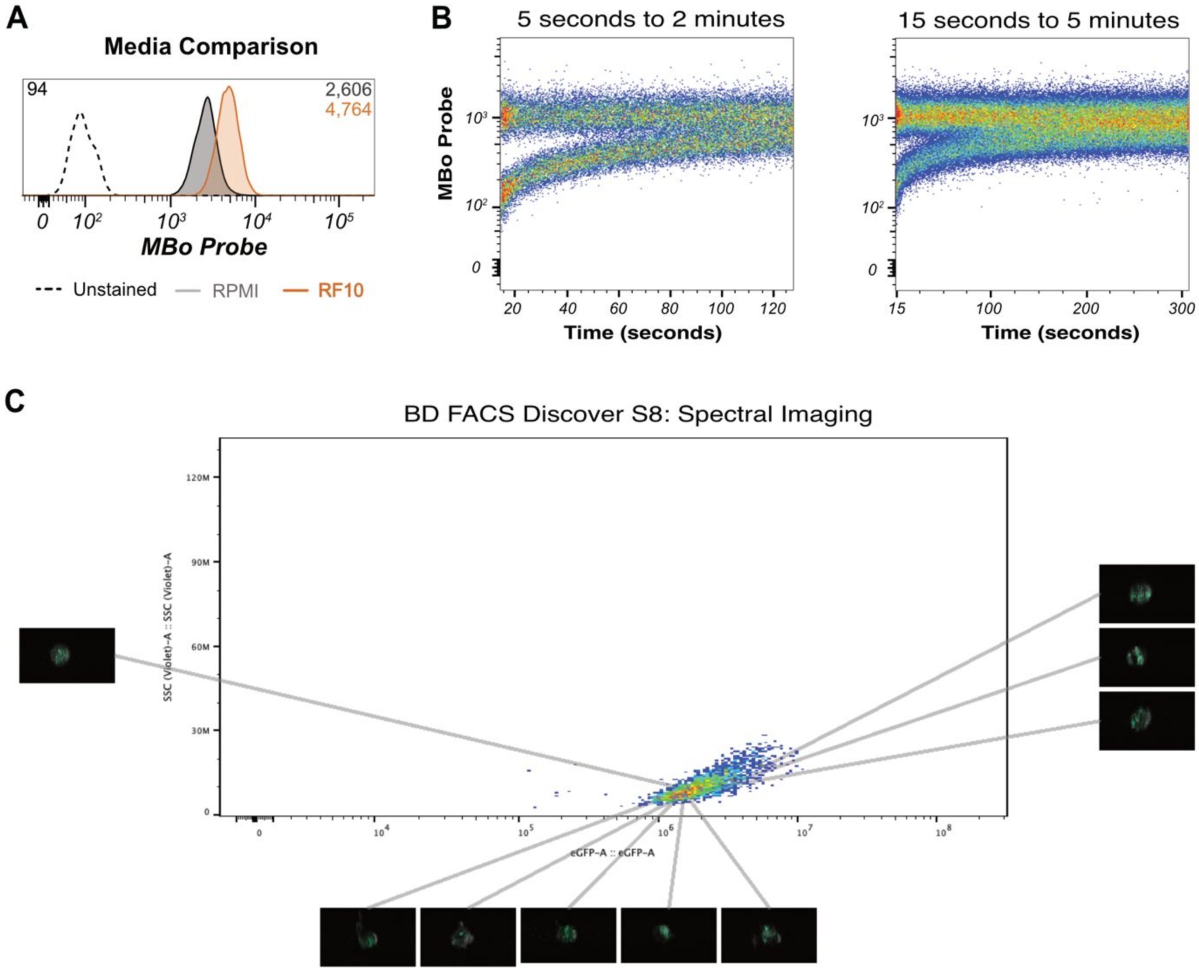

Figure S5 The MBo probe freely diffuses across the plasma membrane and stains intracellular MG. (A) Intracellular fluorescence of the MBo probe in unfixed THP-1 cells measured by flow cytometry. Histograms show detection of endogenous MG in cells cultured for 2 h in serum free media RF0 (grey shaded histogram), serum supplemented media RF10 (orange histogram), and the unstained control (dashed trace). Samples were resuspended in 5 μM of the MBo probe in PBS, stained for 1 h, washed once and resuspended in PBS for flow cytometry. Numbers shown on the histogram represent the gMFI of each population. **(B).** Unfixed THP-1 cells were prepared for endogenous detection of MG as described in (A). After staining, sample replicates were mixed 1:1 with unstained cells and acquired on a flow cytometer for up to 5 minutes, as indicated. Plots show MBo probe staining intensity over time (seconds) during sample acquisition. **(C)** Forward and side scatter profile of the nine spectral images of THP-1 cells shown in Figure 4D, highlighting their representation of the total population.
